# Infants' object location and identity processing in spatial scenes: an ERP study

**DOI:** 10.1002/brb3.184

**Published:** 2013-10-11

**Authors:** Anne H van Hoogmoed, Danielle van den Brink, Gabriele Janzen

**Affiliations:** 1 Radboud University Nijmegen, Behavioural Science InstitutePO Box 9104, Nijmegen, 6500 HE, The Netherlands; 2 Radboud University Nijmegen, Donders Institute for Brain, Cognition, and BehaviourPO Box 9101, Nijmegen, 6500 HB, The Netherlands

**Keywords:** ERP, infants, object processing, spatial cognition

## Abstract

**Background:**

Fast detection and identification of objects in an environment is important for using objects as landmarks during navigation. While adults rapidly process objects within an environment and use landmarks during navigation, infants do not routinely use distal landmarks below the age of 18 months. In the current event-related potential (ERP) study we adopted an oddball paradigm to examine whether infants are capable of processing objects in environments, which is a prerequisite for using objects as landmarks.

**Methods:**

We measured the electrophysiological correlates and time courses related to the processing of changes in object location, object identity, and a switch of two objects.

**Results:**

Twelve-month-old infants showed an Nc (negative central) effect reflecting increased attention likely caused by initial change detection within 300 msec for all three manipulations. In addition, they showed conscious processing of an object change and a location change as evidenced by a positive slow wave (PSW).

**Conclusion:**

This study is the first to show that infants are capable of rapidly detecting changes in single objects when these are presented in an environment, but lack conscious detection of a switch. These results indicate that 12-month-old infants as yet lack the ability to rapidly bind the identity and location of multiple objects within an environment.

## Introduction

The ability to recognize objects and link them to specific locations is crucial in everyday life, from remembering where you left your keys, to finding your way home based on unique objects in the environment. Adults have been shown to make use of distinct objects in the environment, referred to as landmarks, in navigation (for an overview, see Baumann et al. 2010). However, under the age of 18 months children do not routinely make use of distal landmarks to retrieve hidden objects (Newcombe et al. 1998; Balcomb et al. 2011). This may be due to difficulties in individuating and identifying multiple objects in an environment.

A large body of literature has investigated the development of object individuation and identification in infants. Many studies have shown that infants are able to individuate objects based on location at an earlier age than based on identity (Xu and Carey 1996; Newcombe et al. 1999; Tremoulet et al. 2000; Wilcox and Schweinle 2002; Oakes et al. 2006; Krøjgaard 2007). However, Mareschal and Johnson (2003) showed that results can differ based on the type of stimuli used. By the age of 9 months, infants are able to individuate objects both on the basis of their location as well as on the basis of their identity (Wilcox and Schweinle 2002; Káldy and Leslie 2003; Oakes et al. 2006). These processes appear to recruit different brain regions, with location being processed in the dorsal stream and object being processed in the ventral stream (Ungerleider and Mishkin 1982). To detect a switch of two objects, information processed in the dorsal stream needs to be integrated with information processed in the ventral stream. This feature-location binding in working memory is thought to depend on the hippocampus (Káldy and Sigala 2004; Postma et al. 2008).

Research has shown that under certain conditions, young infants are already capable of binding feature (color or shape) and location information. For instance, Oakes et al. (2006, 2009) found that 7-month-old, but not 6-month-old infants were able to individuate an object based on its color and its specific location. Similarly, Káldy and Leslie (2003) showed that 9-month-old infants can individuate objects based on shape and location. However, even though in the latter study infants were shown to be capable of keeping two objects in memory, neither Káldy and Leslie, nor Oakes et al. could dissociate between infants noticing a new object appear at a single location previously occupied by another object and noticing two previously presented objects switching location. The latter finding would provide evidence that children not only are able to keep more than one object in memory, but moreover, that they are capable of binding the respective locations to these multiple objects.

Building on these findings, in this study we investigated 11- to 12-month-old infants' ability to detect changes in one object's location, one object's identity, and a location switch of two objects within an environment. Measuring electroencephalograms (EEG) enabled us to investigate the time course and electrophysiological correlates related to the detection of these three types of object-location changes, and the potential functional differences between the processing of a change in object location, a change in object identity, and a switch in position of two objects.

**Figure 1:**
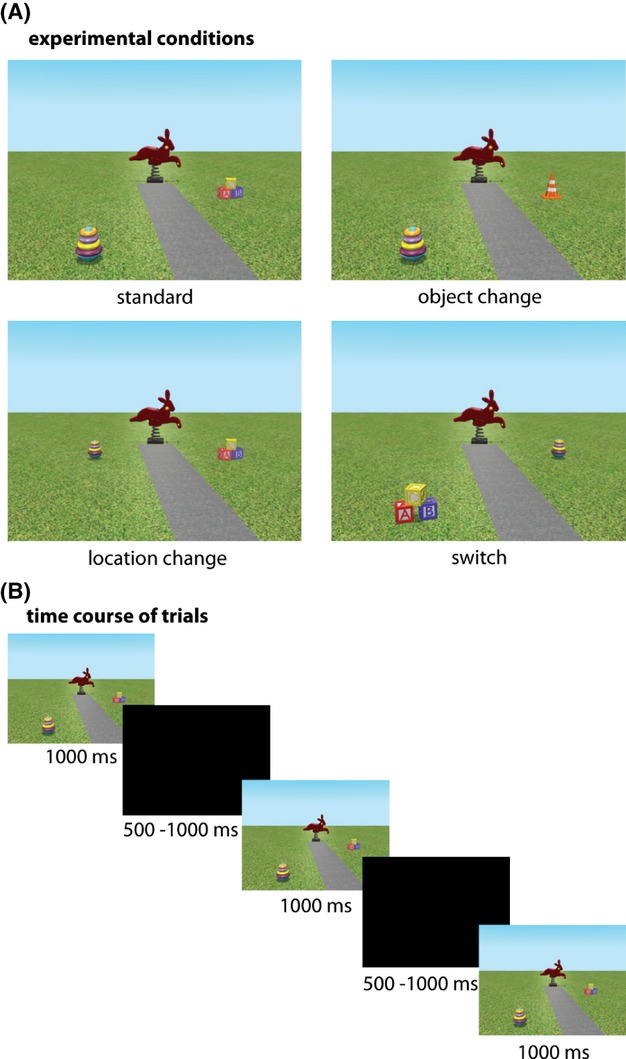
Experimental setup. (A) Exemplars for all conditions within an environment. (B) Time course of the trials in the experiment.

Previous event-related potential (ERP) research on visual perception in infants has primarily focused on face processing (De Haan and Nelson 1997, 1999; Key et al. 2009; Peltola et al. 2009; Parise et al. 2010), although some studies have also investigated object processing (De Haan and Nelson 1999; Bauer et al. 2003). Most of these studies made use of an oddball paradigm, and reported a larger fronto-central negativity starting around 400–600 msec for the oddball stimuli as compared to the standard stimuli in children from 4 weeks to 30 months old (Karrer and Monti 1995; Goldman et al. 2004; Reynolds and Richards 2005; Ackles and Cook 2007; Izard et al. 2008). This negative shift is labeled the Nc (negative central) effect. Two interpretations of the effect are prominent in the literature. On the one hand, many researchers interpret the Nc effect as reflecting a difference in general attentional response (Richards 2003; Ackles 2008; Richards et al. 2010). On the other hand, researchers interpret the effect as reflecting conscious change detection (De Haan and Nelson 1997, 1999; see De Haan 2007 for an overview). The Nc component has not only been found in oddball paradigms but also in paradigms in which familiar and unfamiliar toys were presented with equal frequency (De Haan and Nelson 1997, 1999). Moreover, while the polarity of the Nc effect (deviant minus standard) is often found to be negative, some researchers have also found positive Nc effects (De Haan and Nelson 1997, 1999; Stets and Reid 2011). In several infant studies, the Nc is followed by a positive slow wave (PSW) (Nelson et al. 1998; Richards 2003), which is thought to reflect updating of memory representations of partially encoded stimuli (Nelson and Collins 1992; Hoehl et al. 2012). This means that the representations of new stimuli are strengthened to arrive at a better memory representation. Thus, these studies support the behavioral findings that infants can detect changes in object identity already from at least 9 months of age. However, to date, little is known about the time course of processing object location or the binding of object location and identity in infants. Given the significance of wayfinding in our daily life, information about changes in the environment should be detected rapidly to guide ongoing behavior. ERPs are well-suited to investigate the temporal characteristics of processes involved in object change detection. In the current ERP study, we investigated the time course of several types of object-related changes within an environment. Using an oddball paradigm we presented a standard stimulus in 70% of the trials, and the three oddball stimuli in 10% of the trials each, while measuring the infant's EEG. The oddball stimuli reflected a change in object location (location change), a change in object identity (object change), or a switch in position of two objects (switch) (Fig. 1A).

Previous ERP research investigating object processing in an environment in adults revealed different ERP responses to a change in object location as compared to a change in object identity (Van Hoogmoed et al. 2012). In a delayed match-to-sample task, a location change of an object was detected earlier than a change in object identity. Moreover, a location change elicited a posterior N2 and a central P3 response, whereas a change in object identity elicited an anterior N3 response. Additionally, a switch of two objects was detected even later and only elicited a P3 response. These results support the theory that different neural generators underlie the detection of these changes (e.g. Ungerleider and Mishkin 1982).

In this study, our first objective was to investigate whether infants are capable of fast detection of a location change, an object change, and a switch of two objects in a visual scene. Secondly, we were interested in the ERP signatures related to these changes. On the basis of earlier findings in infant ERP studies, we expected the object change to elicit an Nc effect (Karrer and Monti 1995; Goldman et al. 2004; Reynolds and Richards 2005; Ackles and Cook 2007; Izard et al. 2008). For the location change and switch, we expected either the same Nc component reflecting increased attention and general change detection, or different components following results obtained in adults (Van Hoogmoed et al. 2012). In addition, we hypothesized that the Nc effect would be followed by a PSW effect in either some or all of the oddball conditions, reflecting the updating of the memory representations of the objects in the scene (Nelson and Collins 1992; Hoehl et al. 2012).

## Method

### Participants

In total, 39 healthy 11- to 12-month-old infants participated in the study. All infants were born full term (between 38 and 42 weeks of gestation). Twenty-two infants were excluded from the sample, because of unwillingness to wear the EEG cap, or contributing too few artifact-free trials due to fussiness or excessive movement. The final sample consisted of 17 infants (nine girls, eight boys) with a mean age of 358 days (SD = 5.75). Parents gave their informed consent before the start of the study and were told that participation could be terminated at any time. This study was approved by the local ethics committee (Commissie Mensgebonden Onderzoek Arnhem-Nijmegen, The Netherlands).

### Stimuli

The stimuli consisted of four computer-generated environments created with Blender (http://www.blender.org), consisting of a park, beach, square, or a snow landscape. Each of these environments contained two moveable toy objects, next to one stable object in the middle of the scene and a path leading to this object (see Fig. 1A). For each environment, four different scenes were created. One of these scenes functioned as the standard stimulus, with the three oddball scenes differing from this standard across three conditions. In the object change condition, one of the toy objects in the scene was replaced by another toy object. In the location change condition, one of the toy objects changed position. In the switch condition, the two toy objects switched positions (see Fig. S1 for all stimuli). The position of the toy objects in the different conditions was counterbalanced across environments.

### Procedure

Infants were seated in a car seat in a sound-attenuated booth of 2 × 2 m. They were placed 60–70 cm from the computer monitor and one of the parents was seated behind the child. Parents were asked to sit quietly and not to interact with their child unless the child got upset. The experiment consisted of eight blocks of 50 trials. Each block started with a familiarization phase in which the infants were familiarized with the three objects that would appear in the block. For each object, a short movie of 10 sec was shown in which the object was presented on a white background and moved and rotated to enable the infant to perceive the three-dimensionality of the object. The three videos were presented in random order. If the infant did not attend to the screen during the presentation of the video, the video of this particular object was shown again. After the familiarization phase, the test trials were presented. An oddball paradigm was used in which the standard scene was presented in 70% of the trials, a location change in 10% of the trials, an object change in 10% of the trials, and two objects switching location in 10% of the trials. The stimuli were presented for 1000 msec, followed by a black screen with a random duration of 500–1000 msec (Fig. 1B). The stimuli were pseudo randomized such that the block always started with at least three standard stimuli and an odd stimulus was always preceded by at least two standard stimuli. When the infant looked away from the screen, one of 10 attention grabber movies was played. These attention grabbers were short movies with sound to attract the attention of the infant back to the screen. After the attention grabber, the presentation of trials continued, starting with three standard stimuli. The order of presentation of blocks was counterbalanced across subjects. The experiment ended after eight blocks, but was terminated earlier if the infants showed signs of fussiness. The experimental session was video-recorded and coded offline to exclude trials in which the infant did not attend to the screen.

### EEG recordings and analysis

EEG data were recorded with a 32-electrode actiCAP (Brain Products GmbH, Gilching, Germany) referenced to FCz. Signals were passed through a BrainAmp DC amplifier (Brain Products GmbH) and were recorded online with a sampling rate of 500 Hz. Measured activity was filtered online using a 200 Hz low-pass filter, and a time constant of 10 sec. Impedance was kept below 20 kΩ, which is a standard setting in active electrode recording (Kimura et al. 2010; Junge et al. 2012; Van Elk et al. 2012). After recording, EEG signals were imported into the Matlab-based Fieldtrip toolbox (Oostenveld et al. 2011). Signals were first detrended and then filtered with a 0.5–30 Hz band-pass filter and re-referenced to the mean of the left and right mastoids (Karrer and Monti 1995; Richards 2003; Ackles and Cook 2007; for a review see Hoehl and Wahl 2012). However, due to noisy data on one of these mastoids, for four infants the signal was re-referenced to the right mastoid only, and for two other infants the signal was re-referenced to the left mastoid only. Based on the videos, parts of the data in which the infant did not attend to the computer screen were removed. EEG data were segmented per condition from 200 msec before to 1500 msec after the onset of the stimulus. Segments were baseline corrected by subtracting the mean amplitude in the −100 to 0 msec prestimulus interval. Next, the segments were manually screened for artifacts at all sensors except for those in the outer ring of the cap. Segments were removed when the signal of more than two electrodes exceeded the values of −150 and 150 μV, when the signal jumped more than 75 μV within 5 msec, and when the range of the signal was larger than 75 μV in the baseline period. Whenever a channel deviated substantially from the other channels in more than eight trials while the signal in other channels did not contain artifacts in these trials, this channel was marked as a bad channel. Bad channels were reconstructed based on a linear combination of surrounding channels on the raw data (bad channels were never neighboring channels). After channel reconstruction, segmentation and following steps were repeated on the complete dataset. Averages were based on artifact-free trials. In the standard condition, a mean of 110 trials per subject were included. In the location change condition 12.06 (SD 3.77) trials were included, in the object change condition 12.35 (SD 3.76) trials were included and in the switch condition 12.88 (SD 3.76) trials were included, which was sufficient for computing a reliable ERP, assessed by the visual evoked potential on the occipital Oz electrode (see Fig. S2). Based on previous research, the Nc was analyzed in the 300–700 msec time window in a fronto-central region of interest. Based on visual inspection, a later time window showing a PSW from 700 to 1200 msec was analyzed using the same region of interest. Data were analyzed with repeated measures analysis of variances (ANOVAs) on the mean amplitude values with the within-subject factors Condition (standard, location change, object change, switch) and Electrode (Fz, FC1, FCz, FC2, Cz). Greenhousse-Geisser correction for nonsphericity (Greenhouse and Geisser 1959) was applied whenever appropriate. Corrected *P* values are reported along with original degrees of freedom.

## Results

**Figure 2:**
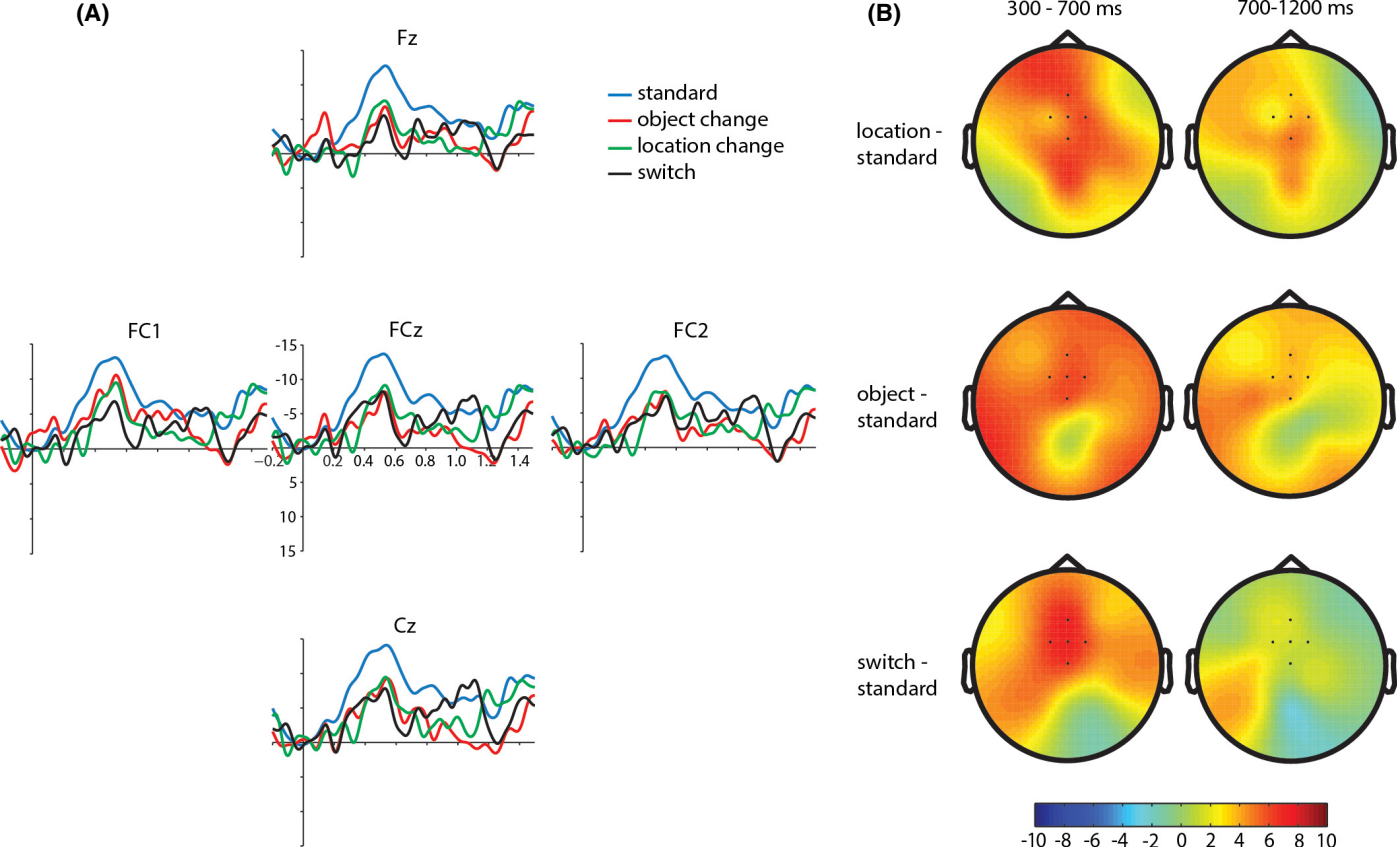
Event-related potential (ERP) data. (A) Grand average waveforms at the five fronto-central electrodes for all conditions. (B) Scalp distributions of ERP effects (change minus standard) in the 300–700 msec and 700–1200 msec time windows.

Figure 2A shows the waveforms at the five fronto-central electrodes included in the analyses and Figure 2B shows the topographical distribution of ERP effects across the scalp. A fronto-central negativity (Nc component) was elicited in all conditions between 300 and 700 msec, which was larger in the standard condition than in the other conditions. The waveforms in the oddball conditions included ∼12 trials, and the waveforms in the standard condition contained 110 trials. The reason for including all trials in the standard condition was to establish a solid baseline with maximized signal-to-noise ratio to compare the oddballs to. To show that the size of the Nc component was not affected by the difference in number of trials included in the averages, Figure S3 shows the standard including all trials as compared to the standard including ∼12 trials, an amount equal to what was used the oddball conditions. An ANOVA in the 300–700 msec time window with the factors Condition and Electrode confirmed the finding of the Nc effect. The results showed a main effect of Condition (*F* (3,48) = 4.41, *P* = 0.008), an effect of Electrode (*F* (4,64) = 6.95, *P* < 0.001), and no interactions (*F* (12,192) = 1.05, *P* = 0.390). Location change, object change, and switch all elicited a smaller negativity than the standard, resulting in a positive effect relative to the standard in this time window (Fig. 2B). A priori contrasts revealed that this effect was significant in all conditions: location change versus standard (*F* (1,16) = 9.77, *P* = 0.007), object change versus standard (*F* (1,16) = 12.76, *P* = 0.003), and switch versus standard (*F* (1,16) = 17.75, *P* = 0.001). In the 700–1200 msec time window, a PSW was elicited in the object change condition and location change condition, while the switch condition did not deviate from the standard in this latency window. The ANOVA revealed no significant effects of Condition and Electrode, and no interaction (all *F* < 1.44, n.s.). However, a priori contrasts showed that the object change and location change differed significantly from the standard (*F* (1,16) = 4.92, *P* = 0.041, *F* (1,16) = 4.55, *P* = 0.049 respectively), whereas the switch did not (*F* (1,16) <1, n.s.).

## Discussion

This study was designed to investigate the ability of 11- to 12-month-old infants to quickly detect object-location changes in a visual scene. EEG was measured during the presentation of an oddball paradigm with a standard stimulus, a stimulus with a location change, a stimulus with an object change, and a stimulus with a switch of two objects to investigate the time course and ERP components related to the processing of these changes. Results show an Nc effect between 300 and 700 msec in all oddball conditions, reflecting either increased attention or conscious change detection (see De Haan 2007 for an overview). Therefore, the Nc effect in all three deviant conditions reveals that the infant brain is capable of detecting a change causing increased attention within this brief time frame. This is crucial evidence that the brain processes are in place for infants to notice a change in the objects' configuration. However, the early detection of these changes may not be conscious and may not include knowledge on what specific change has taken place.

With regard to the observed Nc effect, the effect was the result of a smaller Nc in the oddball conditions as compared to the standard condition. In most infant ERP studies the Nc effect is reversed, showing a larger Nc in oddball conditions as compared to the standard condition (Reynolds and Richards 2005; Webb et al. 2005; Ackles and Cook 2007; Ackles 2008). However, in line with our results, De Haan and Nelson (1997, 1999) also report conditions with larger Nc's for familiar objects and faces than for unfamiliar objects and faces. More recently, Stets and Reid (2011) investigated the effect of the number of trials included in the ERP on the amplitude of the Nc effect. They found a negative effect when all trials (between 11 and 37 trials) were included in the ERP, but a positive effect when only seven trials per condition were included. The polarity of the effect was thus affected by the number of trials included in the analysis. This may account for the reversed effect in our study, as the oddball ERP waveforms included a minimum of seven with a mean 11–13 trials. In this study we maximized the signal-to-noise ratio in the standard condition by including more trials in the EEG average (with a mean of 110 trials). However, Figure S3 clearly shows that the size (and polarity) of the Nc effect was not affected by the inclusion of more trials in the standard condition with respect to the deviant conditions.

In addition to the Nc effect for all manipulations, a subsequent PSW effect was found in the object change and location change conditions as compared to the standard condition. The effect was not found in the switch condition. This result shows that in the latency range of 700–1200 msec after the onset of the stimulus, a change in location and a change in identity are consciously processed as being different from the standard stimulus causing updating of the memory representation for the new stimulus, whereas no evidence was found for conscious processing in the switch condition. Moreover, it suggests that the objects in the scene are processed as separate objects in specific locations. The PSW effect differed for the object change and location change as compared to the switch, while the Nc indicated a similar initial response to the object change, location change, and switch. If the stimuli would have been processed as complete pictures, the similar levels of attention during the Nc period would likely have led to a similar PSW in all oddball conditions. However, the PSW was only present when either a new object was placed into the scene, or a new location was occupied indicating that infants process the objects in the scene as separate objects. The ability of infants to process objects on a computer screen as separate objects opens up the possibility to use computerized environments for studying more complex use of objects, for example landmark use, in infants.

The elicitation of an identical Nc component in all oddball conditions and a similar PSW in the location change and identity change conditions differs from findings in research on adult object processing showing different ERP effects for location change, object change, and switch (Van Hoogmoed et al. 2012). The differently distributed N2 and N3 effects for location change versus identity change in adults suggest that location and identity of objects are processed in distinct brain regions. This finding is in line with the theory of Ungerleider and Mishkin (1982) on the segregation of the dorsal and ventral stream. Many studies have provided evidence for a structural or functional segregation (Tanaka et al. 1991; Haxby et al. 1994; Ungerleider and Haxby 1994; Duhamel et al. 1997; Munk et al. 2002; Pihlajamaki et al. 2005; Jackson et al. 2011), while some contradictory evidence has also been found (Sereno and Maunsell 1998; Op de Beeck and Vogels 2000; Jellema et al. 2004; Cichy et al. 2011). The dorsal/ventral distinction has been a key element in theories on object processing in infancy (Leslie et al. 1998; Mareschal et al. 1999; Schlesinger 2006) and both streams have been shown to be developed already in 5- to 7-month-old infants (Wilcox et al. 2010). Our results reveal similarly distributed Nc effects in response to all manipulations and similar PSW effects to both object and location change, which may imply immaturely developed visual pathways in the infant brain, contradicting the theories on infants' object processing. However, whereas in adults different scalp distributions suggest the involvement of different underlying neural generators, a similar distribution for all conditions in infants does not necessarily imply a contribution of identical neural generators. In general, sources of EEG signals are difficult to localize because of the inverse problem and difficulties in estimating the conductivity of the skull (Wang and Ren 2013). In infants, source localization is even more difficult. The Nc was most prominent at the fronto-central sensors, which coincides with the location of the anterior fontanel. The fontanel is known to produce inhomogeneity in skull conductivity in infants, which causes EEG signals to be distorted (Flemming et al. 2005; Roche-Labarbe et al. 2008; Reynolds and Richards 2009). Because the fronto-central sensors cover the part at which the skull is not closed yet, it is likely that the activity is strongest at this location, regardless of where the signal was generated. Therefore, we cannot make any claims on the underlying neural generators in infants.

Our findings are in line with previous research showing that changes in object location and in object identity are detected early in life (Wilcox and Schweinle 2002; Káldy and Leslie 2003, 2005; Oakes et al. 2006). The lack of conscious detection of the switch could be due to the maturation of the brain mechanisms involved in binding object location to object identity. In adults, functional magnetic resonance imaging (fMRI) studies have shown that feature-location binding is dependent on the hippocampus (Piekema et al. 2006; Hannula and Ranganath 2008). The hippocampus is a brain structure subject to protracted development throughout childhood (Gogtay et al. 2006; Lavenex and Banta Lavenex 2013). Our finding that object location and object identity, but not a switch of two objects is consciously detected could be due to the immaturity of the hippocampus. Alternatively, it is also possible that 12-month-olds are capable of binding multiple objects to their respective locations, but that they were unable to do so in our experiment as a result of the rapid presentation of the scenes. It is possible, that given more time, infants would show evidence of feature-binding of multiple objects within an environment. Therefore, more research is needed to clarify the development of the hippocampus and its role in object-location binding in infants, as well as the effect of speeded presentation on object-location binding processes in infants.

To conclude, this study is the first to cohesively show that 12-month-old infants are capable of rapidly processing changes in objects and changes in location when objects are presented in a contextually rich environment. The use of EEG enabled us to demonstrate that they show increased attention based on initial change detection amazingly fast, already within 300 msec. In addition, we have shown that they consciously process object changes and location changes further to strengthen their memory representations. Moreover, our results show that 12-month-old infants do not yet show fully developed object processing or scene memory, as they do not show conscious processing of two objects switching positions which requires object-location binding of multiple objects. While infants have been shown to be able to bind object and location in other studies (Káldy and Leslie 2003), it seems that they are not yet fully capable of quickly recognizing and remembering more objects in specific locations. The ability to quickly bind multiple objects to specific locations within an environment is a prerequisite for using landmarks during navigation. Therefore, young infants' incapability to successfully use landmarks (e.g. Newcombe et al. 1998; Balcomb et al. 2011) may be the result of an inability to process multiple objects in an environment. Alternatively, the delay in landmark use as compared to object recognition could be caused by the infants' inability to retain object information in memory over time (Richmond and Nelson 2007). Computerized environments can be used to investigate whether the prolonged development of memory for objects causes the delay between the detection of object changes and the use of landmarks in navigation or whether this delay is related to the later onset of fast detection of binding objects to specific locations within an environment.

## Supporting Information

**Figure S1.** Complete stimulus set in each environment.

**Figure S2.** Grand average waveform at Oz for all conditions after onset of stimulus, showing onset and offset visual evoked potentials.

**Figure S3.** Grand average waveforms at the five fronto-central electrodes for the standard condition showing no difference in amplitude when different number of trials was included in the waveform. The blue line represents the standard as used in the analysis. The red line represents the mean of all standards that were directly followed by an odd stimulus. The green line includes every third presentation of a standard followed by an odd stimulus, to match the number of trials in the odd conditions.
