# Supplementary figures and images for: Infants' object location and identity processing in spatial scenes: an ERP study
Source: Brain Behav. 2013 Oct 11;3(6):729–37. doi: 10.1002/brb3.184 (PMC3868177; doi:10.1002/brb3.184)

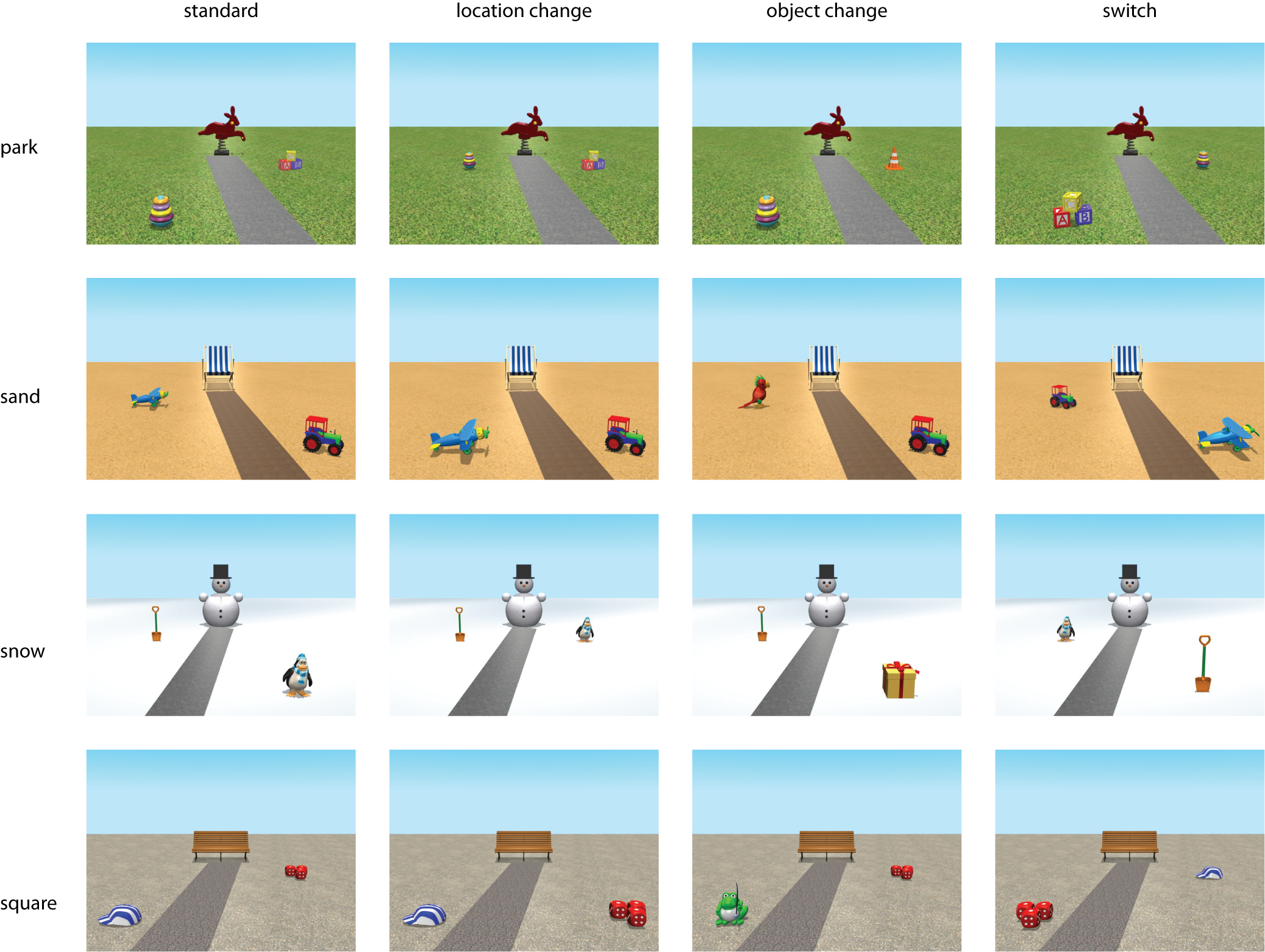

Supplement: Supplementary file 1 [file brb30003-0729-SD1.tif]

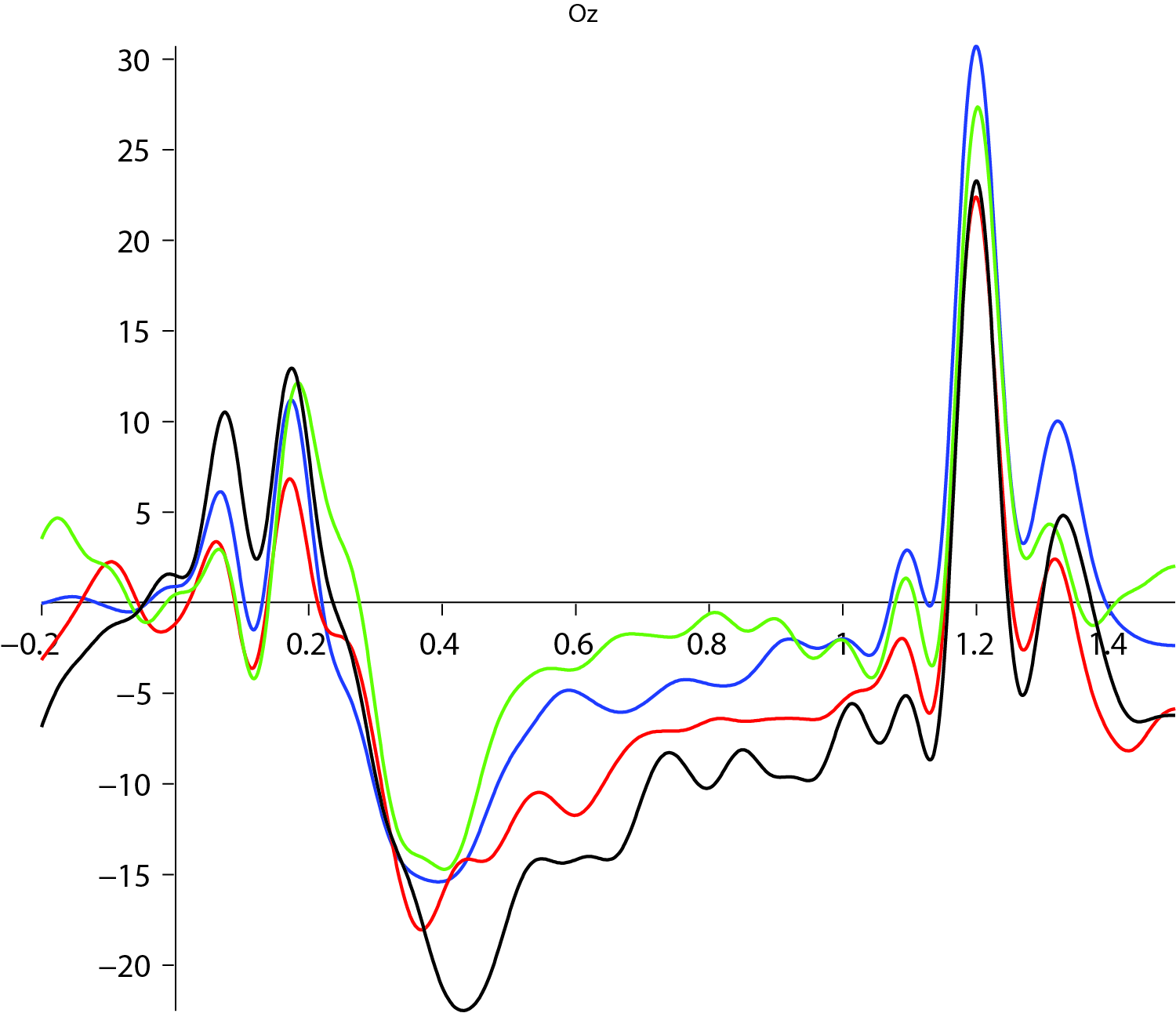

Supplement: Supplementary file 2 [file brb30003-0729-SD2.tif]

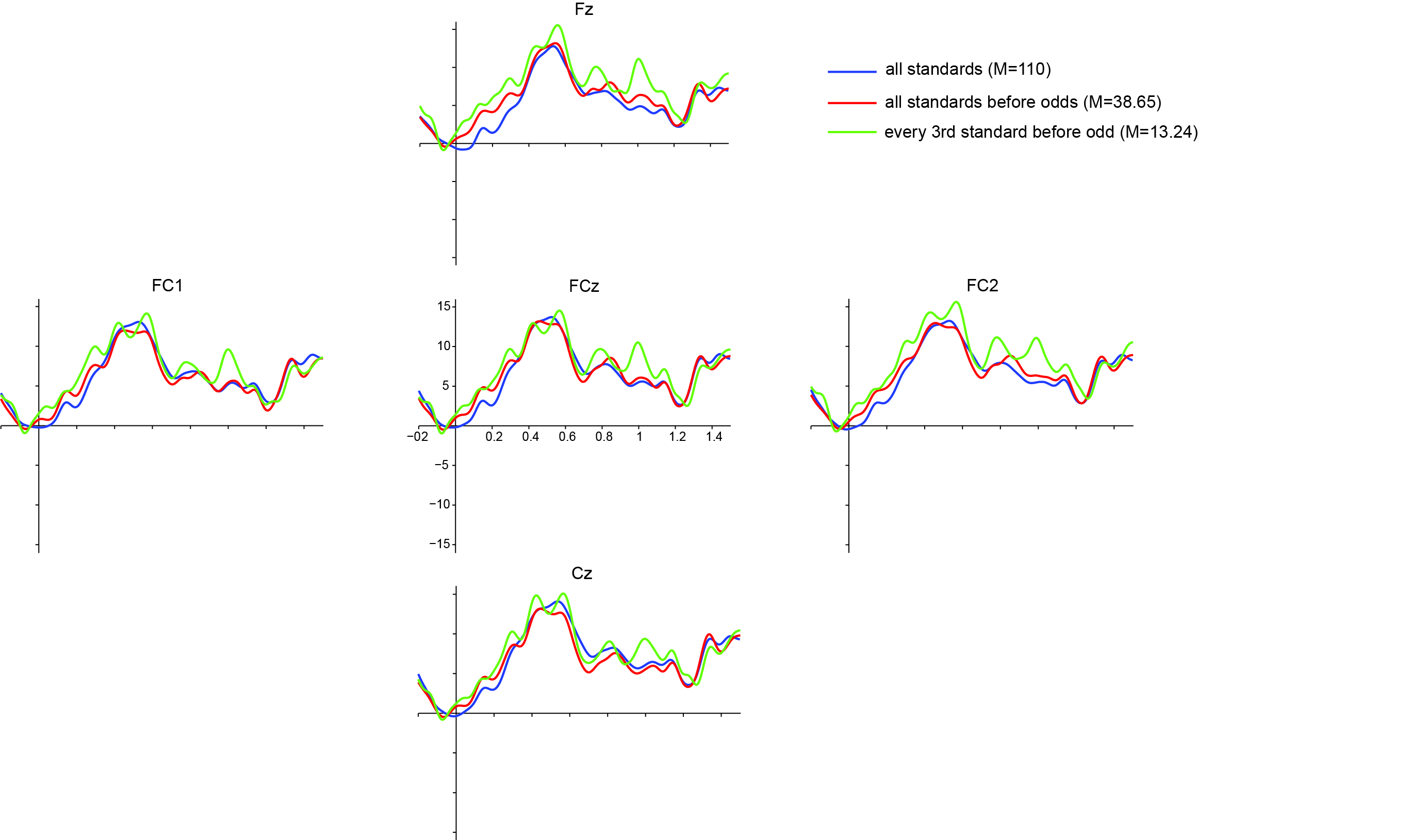

Supplement: Supplementary file 3 [file brb30003-0729-SD3.tif]
